# New Appliances and Things Medical

**Published:** 1906-03-03

**Authors:** 


### NEW APPLIANCES AND THINGS MEDICAL.

[We shall be glad to receive at our Office, 28 & 29 Southampton Street, Strand, London, W.C., from the manufacturers, specimens of all new preparations and appliances which may be brought out from time to time.]

### MOSELEY'S COCOA.

(Blended and Prepared by Foods, Limited, Stockport.)

We have received a sample of this cocoa, which appears to fulfil all the conditions which should be demanded of a good cocoa. It forms a pleasant beverage, whether made with milk or water, and has as much right to be called a soluble preparation of cocoa as any of which we are aware. We are pleased to have received this specimen as it gives us an opportunity of discoursing upon cocoa as a beverage. At the present time, in view of the enormous and increasing consumption of tea in this country, a few remarks upon this subject may not be out of place. Tea is essentially a beverage for the rich in that it stimulates, but does not nourish, or in other words, helps the organism to unlock energy, but does not supply it. It is said of Dr. Johnson that his tea-kettle never had time to cool; but Dr. Johnson's diet doubtless contained ample sources of nourishment, unlike that of the tea-drinking poor of to-day. If we look carefully at the chemical composition of cocoa we find that it not only contains, as tea and coffee, a stimulating alkaloid, namely, theobromine, but also a considerable quantity, namely, about 25 per cent. of fat and approximately 17 to 20 per cent. of nitrogenous matter. Exact physiological experiments made with cocoa have shown that it does not effect the processes of digestion to the same extent as tea or coffee. The natural cocoa-seed, from which cocoa by a more or less complicated process is made, contains a much greater proportion of fat than the finished product. In no class of food is digestive idiosyncrasy so manifest as in fats, and especially does this seem true of the vegetable fats. Hence there are some patients, who in spite of the high nutritive value of cocoa, are not able to take it, or can only take preparations of it in which the fat is reduced to a minimum. As a stimulant ordinary cocoa is about as active as tea or coffee—a teacupful of the former containing about the same quantity (1.5 grains) of theobromine as the latter of caffeine. The nitrogenous constituents of cocoa are not so important from the standpoint of nutrition as they seem because a considerable quantity of the 20 per cent. of these substances contained in cocoa consists of so-called amido-bodies, which chemically and physiologically are allied to theobromine. We think cocoa as a beverage is strongly to be recommended, and that the preparation before us is a good one.

### VASELINE.

(Chesebrough Manufacturing Company, 42 Holborn Viaduct, London, E.C.)

We have received four samples of this company's preparations—namely, ordinary vaseline, white vaseline, carbolated vaseline, and capsicum vaseline. Vaseline is a semi-solid mixture of various hydro-carbons obtained during the distillation of petroleum. It possesses many advantages over the animal and vegetable fats as an emollient, the chief perhaps being that it will keep indefinitely without becoming rancid, and possesses, like the allied body liquid paraffin, considerable solvent power for certain antiseptics. The preparations before us are excellent, and we notice especially the combination of vaseline with capsicum, the so-called capsicum vaseline. This compound we think practitioners will find most useful as a rubefacient.

